# The combination of methylsulfonylmethane and tamoxifen inhibits the Jak2/STAT5b pathway and synergistically inhibits tumor growth and metastasis in ER-positive breast cancer xenografts

**DOI:** 10.1186/s12885-015-1445-0

**Published:** 2015-06-19

**Authors:** Nipin SP, Pramod Darvin, Young Beom Yoo, Youn Hee Joung, Dong Young Kang, Don Nam Kim, Tae Sook Hwang, Sang Yoon Kim, Wan Seop Kim, Hak Kyo Lee, Byung Wook Cho, Heui Soo Kim, Kyung Do Park, Jong Hwan Park, Soung Hoon Chang, Young Mok Yang

**Affiliations:** 1 Department of Pathology, School of Medicine, and Institute of Biomedical Science and Technology, Konkuk University, Seoul, 143-701 Korea; 2 Department of Surgery, School of Medicine, Konkuk University, Seoul, 143-701 Korea; 3 Genomic Informatics Center, Hankyong National University, Anseong, Korea; 4 Department of Animal Science, College of Life Sciences, Pusan National University, Pusan, Korea; 5 Department of Biological Sciences, College of Natural Sciences, Pusan National University, Busan, Korea; 6 Department of Preventive Medicine, School of Medicine, Konkuk University, Chungju, 380-701 Korea

**Keywords:** Breast cancer, Methylsulfonylmethane, Tamoxifen, Jak2/STAT5b pathway, Xenograft, Metastasis

## Abstract

**Background:**

Combination therapy, which reduces the dosage intensity of the individual drugs while increasing their efficacy, is not a novel approach for the treatment of cancer. Methylsulfonylmethane (MSM) is an organic sulfur compound shown to act against tumor cells. Tamoxifen is a commercially available therapeutic agent for breast malignancies.

**Methods:**

In the current study, we analyzed the combinatorial effect of MSM and tamoxifen on the suppression of ER-positive breast cancer xenograft growth and metastasis. Additionally, we also validated the molecular targets by which the drug combination regulated tumor growth and metastasis.

**Results:**

We observed that the combination of MSM and tamoxifen regulated cell viability and migration *in vitro*. The intragastric administration of MSM and subcutaneous implantation of tamoxifen tablets led to tumor growth suppression and inhibition of the Janus kinase 2 (Jak2)/signal transducer and activator of transcription 5b (STAT5b) pathway. Our study also assessed the regulation of signaling molecules implicated in the growth, progression, differentiation, and migration of cancer cells, such as Jak2, STAT5b, insulin-like growth factor-1Rβ, and their phosphorylation status.

**Conclusions:**

Study results indicated that this combination therapy inhibited tumor growth and metastasis. Therefore, this drug combination may have a synergistic and powerful anticancer effect against breast cancer.

**Electronic supplementary material:**

The online version of this article (doi:10.1186/s12885-015-1445-0) contains supplementary material, which is available to authorized users.

## Background

Breast cancer (BCa) is one of the most common cancer in women across the world with the second highest rate of mortality each year [1, 2]. Recent studies have proven that environmental factors play a vital role in the incidence of BCa [3]. Cell proliferation and apoptosis regulates the development of cancer, and these two mechanisms are considered as markers for assessing different therapeutic agents [4].

**Table 1:** RT-PCR primers sequences used for the amplification of multiple human cDNAs

Sl No	Gene	Annealing temperature (°C)	Product size (bp)	Sequence (5’ - 3’)
1	Cyclin-D1	58	135	F – gctgcgaagtggaaaccatc
				R – cctccttctgcacacatttgaa
2	IGF-1Rβ	58	522	F – actatgccggtgtctgtgtg
				R – tgcaagttctgattgttgag
3	IGF-1	58	498	F – tcctcgcatctcttctacct
				R – tctggactcgccagtccaat
4	VEGF	58	405	F – aggagggcagaatcatcacg
				R – caaggcccacagggattttc
5	18S	58	490	F – agccttcggctgactggctgg
				R – ctgcccatcatcatgacctgg
6	MMP2	53	665	F – gagttggcagtgcaatacct
				R – gccatccttctcaaagttgt
7	MMP3	60	432	F – cctgctttgtcctttgatgc
				R – tgagtcaatccctggaaagt
8	MMP9	58	455	F – cctgccagtttccattcatc
				R – gccattcacgtcgtccttat
9	hIGF-1 (CHIP assay)	60	700	F – tggcatgttttgaggttttg
				R – gattggttgtgtggcatgag

Janus kinase (Jak), signal transducer and activator of transcription (STAT), and insulin-like growth factor (IGF) are the major genes overexpressed in breast cancer [5]. Upon cytokinebinding to the receptors, Jak tyrosine kinases phosphorylate specific tyrosine residues in the receptors, which then act as docking sites for the STAT family of transcription factors [6]. The STAT family consists of seven different transcription factors that play crucial roles in cytokine signaling [7]. STAT5b, an important member of the STAT family, is activated by phosphorylation, dimerizes, and then translocates to the nucleus where it binds to a DNA response element and directly regulates the expression of target genes [8, 9]. IGF-1, IGF-1Rβ, and cyclin D1 are the main downstream targets of STAT5b [10, 11]. IGF-1Rβ is a transmembrane tyrosine kinase that participates in cell proliferation and apoptosis. Due to its influence on invasion and metastasis, IGF-1Rβ is considered to be an anticancer treatment target [12].

The estrogen receptor (ER) has been shown to be of prognostic significance for BCa patients. More importantly, ER can be a predictive marker for endocrine therapy in the clinical management of BCa [13, 14]. Tamoxifen (Tam) is a selective ER modulator that can act as either an ER agonist or antagonist, and is a synthetic, non-steroidal compound used for the treatment of ERα-positive and other hormonally-responsive BCa [15]. Tam acts by controlling the binding of estradiol to the ER and forms a tam-ER complex which then binds to DNA. This leads to the failure of transcriptional activation and growth inhibition in estrogen-dependent cells [16].

Research efforts to find natural compounds for tumor growth suppression have revealed great potency and potential in cancer management. Methylsulfonylmethane (MSM), also known as dimethyl sulfone, is an organic sulfur compound mainly present in foods such as fruits and vegetables, and in beverages as well. Therefore, MSM intake is possible through diet [17–19]. Study results have demonstrated that MSM was associated with antioxidant and anti-inflammatory mechanisms [20, 21]. The pharmacokinetics studies on MSM indicated that, uptake and distribution of MSM throughout the body rapidly and it was eliminated through the urine [22, 23]. The studies related with high dosage of oral administration of MSM showed the upregulated levels of MSM in blood which indicating the ability of MSM to diffuse in blood even in high concentration [24, 25]. Recently, we suggested that MSM could substantially decrease the viability of human BCa cells due to its anticancer activities, such as contact inhibition, wound healing, and blockage of cell migration [10, 26]. Additionally, Caron et al. reported that MSM manifests anti-cancer activity in metastatic BCa cells [27, 28].

Combination therapy is not a new approach for the treatment of cancer. Its purpose is to reduce the dose intensity in order to mitigate toxicity while increasing the efficacy of the drugs. Our principal aim was to develop a new drug combination that could be more effective with less, or no, toxicity by altering drug concentrations.

MSM has the ability to inhibit STAT3 and STAT5b in human breast cancer cell lines [10]. Tam has already found out to be an anti-cancer drug used in the combination therapy [29–31]. It can also synergize the efficacy of other drug in the combination therapy [32–34]. So in the current study, we hypothesized that the combination of MSM and Tam could synergize the anti-BCa effects of tam at an even milder dose, owing to the ability of MSM to inhibit the STAT5b and STAT3 signalling pathways. Such a drug combination may have the ability to synergize tumor suppression *and* Jak2/STAT5b pathway inhibition.

## Materials and methods

### Antibodies and reagents

Human breast adenocarcinoma, MCF-7, and T47D cell lines were purchased from South Korean Cell Bank (Seoul, KR). RPMI-1640 was purchased from Sigma Chemical (St. Louis, MO, USA). Penicillin-streptomycin solution and fetal bovine serum (FBS) were purchased from Hyclone (South of Logan, Utah, USA). 0.05 % trypsin-ethylenediaminetetraacetic acid was purchased from Gibco-BRL (Grand Island, NY, USA). STAT5b, vascular endothelial growth factor (VEGF), VEGF-R2, IGF-1Rβ, matrix metalloproteinase (MMP)2, MMP3, MMP9 antibodies, and secondary antibodies (goat anti-mouse and rabbit immunoglobulin G [IgG]-horseradish peroxidase) were obtained from Santa Cruz Biotechnology (Santa Cruz, CA, USA). Jak2 was obtained from Millipore (Billerica, MA, USA). Phosphorylated Jak2 antibody were purchased from Cell Signalling Technology (Beverly, MA, USA), and phosphorylated STAT5 was purchased from Upstate Biotechnology (Lake Placid, NY, USA). β-actin was purchased from Signa Chemical Co. (St. Louis, MO, USA). The enhanced chemiluminescence (ECL Plus) detection kit was purchased from Amersham Pharmacia Biotech (Piscataway, NJ, USA). Restore™ Western Blot Stripping Buffer and NE-PER kits were purchased from Pierce (Rockford, IL, USA). RNeasy mini kits and Qiaprep spin miniprep kits were purchased from Qiagen (Hilden, Germany). Reverse transcriptase-polymerase chain reaction (RT-PCR) premix kits and VEGF, IGF-1, IGF-1Rβ, cyclin D1, MMP2, MMP3, MMP9, 18 s primers for RT-PCR were synthesized by Bioneer (Daejon, Korea). Electrophoretic mobility shift assay (EMSA) kits and oligonucleotide probes (STAT5b) were obtained from Promega Corp (Madison, WI, USA). Paraformaldehyde and mounting solution for immunohistochemistry were purchased from Dae Jung Chemicals & Metals Co. (Shineung-city, Korea) and Life Science (Mukilteo, WA, USA). Imprint chromatin immunoprecipitation assay kits, Triton X-100, and tamoxifen were obtained from Sigma Chemical Co. (St. Louis, MO, USA). MSM was purchased from Fluka/Sigma Co. (St. Louis, MO, USA). 17β-estradiol pellets (0.72 mg, 60 days release) and tamoxifen tablets (0.72 mg, 60 days release) were purchased from Innovative Research of America (Sarasota, FL, USA).

### Ethics statement

All procedures for animal experiments were approved by the Committee on the Use and Care on Animals, (Institutional Animal Care and Use Committee, Seoul, Korea) and performed in accordance with the institional guidelines.

### Cell culture and treatment

MCF-7, and T47D cell lines were maintained in RPMI-1640 medium containing 10 % FBS, 100U/mL penicillin and streptomycin at 37 °C in 5 % CO_2_. The cells were placed in airtight chambers (Nu Aire, Plymouth, MN, USA). At the beginning of each experiment, the cells were resuspended in the medium at a density of 2.5 × 10^5^ cells/mL. Cells were treated with Tam at 25 μM, MSM at 300 mM and/or a combination of both (Tam at 15 μM and MSM at 200 mM).

### Cell proliferation inhibition

Cell viability was assayed by measuring blue formazan that was metabolized from 3-(4,5-dimethylthiazol-2-yl)-2,5-diphenyl tetra-zolium bromide (MTT) by mitochondrial dehydrogenase, which is only active in live cells. The cells were resuspended in the medium one day before drug treatment, at a density of 3 × 10^3^ cells per well in 96-well culture plates. Liquid medium was replaced with fresh medium containing dimethyl sulfoxide (DMSO) for control (vehicle). Cells were incubated with various concentrations of Tam, MSM, and their combinations (1:10000, 3:40000). MTT (5 mg/mL) was added to each well and incubated for 4 h at 37 °C. The formazan product formed was dissolved by adding 200 μl DMSO to each well, and the absorbance was measured at 550 nm on an Ultra Multifunctional Microplate Reader (TECAN, Durham, NC, USA). All measurements were performed in triplicate, and were repeated at least three times.

### Apoptosis analysis

Fluorescein-conjugated annexin V (annexin V-FITC) was used to quantitatively determine the percentage of cells undergoing apoptosis. Drug-treated cells were washed and resuspended in binding buffer at a concentration of 1 × 10^6^ cells/mL. The cells undergoing apoptosis were stained with annexin V-FITC and propidium iodide. After incubation for 15 min at room temperature in the dark, the percentage of apoptotic cells was analyzed using flow cytometry (Becton-Dickinson FACScan, San Jose, CA, USA). 10 μM camptothecin was used as the positive control for the analysis.

### Western blotting

The MCF-7 and T47D cell lines were treated with Tam, MSM, and their combination for predetermined periods of time. Whole cells were lysed on ice with radioimmunoprecipitation lysis buffer containing phosphatase and protease inhibitors. Cells were disrupted by aspiration through a 23-gauge needle, and centrifuged at 15,000 rpm for 10 min at 4 °C to remove cellular debris. Protein concentrations were measured using the Bradford method. Equal amounts of proteins were resolved on sodium dodecyl sulfate-polyacrylamide gel electrophoresis (SDS-PAGE) and transferred onto nitrocellulose membrane. The blots were blocked for 1 h with 5 % skim milk. Membranes were probed over night at 4 °C with a primary antibody followed by HRP-conjugated secondary antibodies. Detection was performed using the ECL Plus detection kit and an LAS-4000 imaging device (Fujifilm, Japan).

### Apoptotic DNA ladder analysis

The MCF-7 and T47D cell lines were treated with Tam, MSM, and their combination for 24 h. The cells were then collected by centrifugation, and DNA ladder analyses were carried out using DNA ladder kits. The DNAs were isolated as per kit protocol and products were then analyzed by electrophoresis with 1 % agarose gel containing ethidium bromide. Lyophilized apoptosis U937 cells were used as a positive control.

### RT-PCR

Total RNAs were extracted using RNeasy Mini Kits (Qiagen) and quantified spectrometrically at 260 nm. RT-PCR analysis for IGF-1, IGF-1R, cyclin D1, VEGF, and 18 s RNAs were then performed. cDNA was synthesized from total RNA by RT at 42 °C for 1 h and 80 °C for 15 min using first strand cDNA synthesis kits (Bioneer, Korea). PCR was conducted using cDNA. The PCR conditions consisted of denaturation for 30 s–1 min at 94–95 °C, annealing for 30 s–1 min at 55–60 °C, and extension for 30 s–1 min at 72 °C. PCR products were analyzed by 1 % agarose gel stained with ethidium bromide.

### EMSA

The DNA binding activity of STAT5b was assessed using EMSA, in which a labeled double-stranded DNA was used as a DNA probe to bind active STAT5b proteins in nuclear extracts. Nuclear protein extracts were prepared with a nuclear extract kit (Panomics, AY2002). The EMSA experiment was performed by incubating a biotin-labeled transcription factor-STAT5b probe with treated and untreated nuclear extracts. Proteins were resolved on a non-denaturing 6 % PAGE gel (Bio-Rad, Korea). The proteins in the gel, transferred to a nylon membrane and detected using streptavidin-HRP and a chemiluminescent substrate.

### Chromatin immunoprecipitation assay (ChIP)

A ChIP assay was performed using an Imprint Chromatin Immunoprecipitation Kit (Sigma, St. Louis, MO, USA) according to the manufactures protocol. Briefly, MCF-7 cells were fixed with 1 % formaldehyde and quenched with 1.25 M glycine. After washing with PBS, the cells were suspended in nuclei preparation buffer and shearing buffer, and sonicated under optimized conditions. This sheared DNA was then centrifuged and a cleared supernatant was used for protein/DNA immunoprecipitation. The clarified supernatant was diluted with dilution buffer (1:1 ratio) and 5 μl of diluted samples were removed as an internal control. The diluted supernatant was incubated with antibody (STAT5b) in pre-coated wells for 90 min. For negative and positive control, normal mouse IgG and anti-RNA polymerase II were used, respectively. The unbound DNA was washed off with IP wash buffer and the bound DNA was collected by cross link reversal using DNA release buffer containing proteinase K. The released DNAs and the DNA from the internal controls were purified with GenElute Binding Column G. The DNA was then quantified using conventional PCR.

### Wound healing assay

MCF-7 cells were cultured in 6-well plates at a concentration of 1 × 10^5^ cells/well in RPMI-1640 media and incubated for 24 h in a humidified chamber. After becoming a confluent monolayer, the cell layers were scratched with a pipette tip and washed with PBS to remove cell debris. Cells were treated with the required concentrations of drugs (Tam, MSM, and their combination). Control cells were not treated. Wound edges were photographed at different time intervals using a microscope. The relative area of wound closure was measured using ImageJ software [35] (NIH Image, Bethesda, MD, USA).

### Matrigel invasion assay

The transwell invasion assay was performed with the help of Matrigel pre-coated, ready to use invasion chambers (BD Biocoat, MA, USA). Cells suspended at 5 × 10^4^ were added to the inserts. The drug-containing media was added to the receiver plate and the inserts were placed onto it. After a 24 h incubation in a humidified chamber at 37 °C, the cells that invaded to the apical surface of the inserts were resolved with crystal violet. The cells on the upper surface were removed using a cotton swab and the invaded cells were observed using a microscope. Focus was placed on four distinct areas and the cells were counted.

### Small interference RNA (siRNA) analysis

T47D cells (1 × 10^5^) were cultured on 6-well plates and grown to 50 % confluence. The cells were then transfected with ON-TARGET plus SMARTpool siRNA targeting STAT5b or ON-TARGETplus non-targeting siRNA (Dharmacon, Chicago, IL, USA) using Fugene 6 (Roche, IN, USA) according to the manufacturer’s instructions. Following transfection with this mixture for 24 h, invasion assays were conducted without adding drugs for an additional 24 h. Different areas were captured and the cells were counted.

### Tumorigenecity

All procedures for animal experiments were approved by the Committee on the Use and Care of Animals (Institutional Animal Care and Use Committee, Seoul, Korea) and performed in accordance with institutional guidelines. For the establishment of ER–positive MCF-7 xenografts, mice were ovariectomized and a 17β-estradiol pellet (0.72 mg, 60 days release; Innovative Research of America, Sarasota, FL, USA) was implanted subcutaneously into the neck to facilitate optimal tumor growth. The xenografts were initiated by subcutaneously injecting MCF-7 cells (1 × 10^7^) into the flank of the right hind leg. When tumors reached between 6–8 mm in diameter, 6 mice were randomly assigned to one of four groups: control, Tam, MSM, or their combination. For the MSM-treated group, 3 % MSM was administered as an intragastric injection of 100 μl with triple distilled water. For the Tam-treated group, a Tam pellet (0.72 mg, 60 days release; Innovative Research of America) was implanted subcutaneously into the neck. For the combination-treated group, a Tam pellet was implanted on the neck and MSM was administered as an intragastric injection. The injections were repeated one time per day. Tumor growth was monitored by periodic measurements with digital calipers. When the diameter of tumors reached 2 cm, or after 30 days of treatment, the animals were sacrificed. In our experiments, no mice were observed to be dying due to tumor loading. All available BCa specimens collected from human BCa xenograft mice were reviewed and included in the study. Mice were euthanized and tumors were removed. The tumors were fixed with 4 % paraformaldehyde followed by paraffin embedding and sectioning (5 mm). The sections were stained with hematoxylin and eosin (H&E).

### Metastatic animal models

Orthotopic metastatic animal models were induced by tail vein injection of MCF-7 cells into 5-week-old BALB/c nude mice (Orient Bio, Korea). For inducing tumors in the MCF-7 model, mice were overiectomized and implanted with a 17β-estradiol pellet subcutaneously into the neck. The mice were randomly devided into four goups and treatment was administered as in the xenograft animal model. A Tam pellet was also subcutaneously injected into the neck along with 17β-estradiol. The control group was treated with vehicle, and for the MSM-treated group, 300 mM MSM was administered as a 100 μl intragastric injection. The combination group was treated with 300 mM MSM and a Tam pellet. Treatment was given for 30 days, at which time the mice were sacrificed. Lungs were removed, fixed in 10 % formalin, and paraffin embedded. The analysis of the tissue was performed with the help of H&E staining. The numbers of metastatic tumors on the lung were counted and the relative inhibition of metastatsis was determined.

### H&E staining

Consecutive sections (5 μm thick) were made using the formalin-fixed xenografts and lungs embedded in paraffin. Sections were then deparaffinized and rehydrated with xylene, followed by washing in a decreasing gradient of ethanols (100 %, 95 %, 90 %, 80 %, and 70 %) and staining with H&E. The slides were observed under a microscope and photographed.

### Immunoflurescence (IF)

Formalin-fixed paraffin-embedded breast tumor xenografts were deparaffinized with 100 % xylene, rehydrated in a decreasing gradient of ethanols, permeabilised with 0.1 % triton X-100, and blocked with 10 % normal goat serum in PBS. These were then incubated with the STAT5b or IGF-1Rβ primary antibodies, followed by incubation with the appropriate secondary antibody, Alexa Fluor 594 (rabbit) and Alexa Fluor 488 (mouse) (Invitrogen, CA, USA). For the detection of the nucleus, tissue sections were incubated with fluorochrome 4’-6-diamidino-2-phenylindole for one minute and rinsed with PBS. Samples were observed and photographed under a fluorescent microscope.

### Synergy quantification and statistical analysis

The synergy induced by the drug combination was analyzed with the use of Compusyn software. Combination index (CI) values were computed based on the method of Chou and Talalay [36]. CI computation values to be interpreted as CI > 1 additive effect CI < 1 synergism. All experiments were repeated three times and the results are expressed as mean ± SEM. Statistical analyses were conducted using student’s *t* -tests or *ANOVA* tests with the SAS program.

## Results

### Synergistic inhibition of cell proliferation by the combination of tamoxifen and MSM

**Fig. 1:**
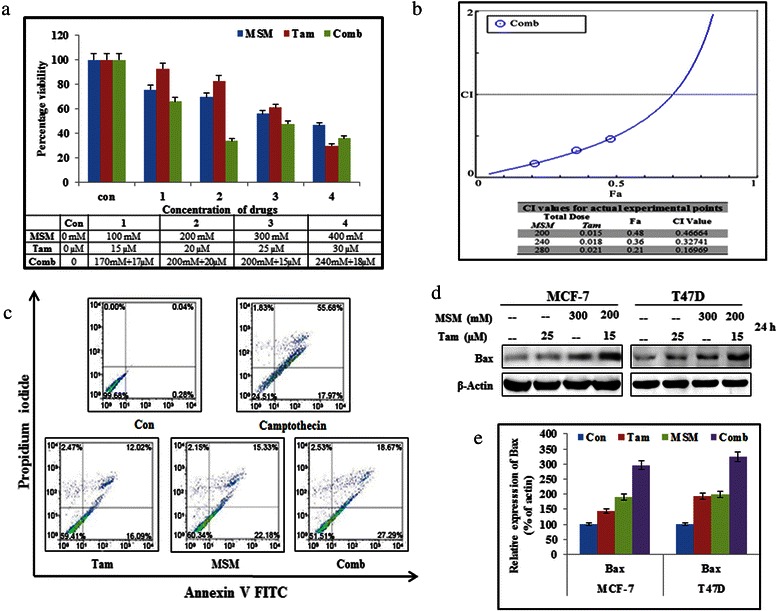
Synergistic inhibition of cell proliferation by the combination of Tam and MSM and induction of apoptosis. (**a**) 3-(4,5-dimethylthiazol-2-yl)-2,5-diphenyl tetra-zolium bromide assay for cell proliferation arrest by Tam, MSM, and their combinations in MCF-7 breast cancer cells. (**b**) Combination index plot for drug combination and table for various concentrations of Tam and MSM by Compusyn. (**c**) Apoptosis induced using MSM, Tam, or the combination in MCF-7 cells quantified using flow cytometry. Camptothecin was used as positive control. (**d**) Western blotting analysis of Bax protein levels in MCF-7 and T47D cells, and after treatment with Tam, MSM, or their combination for 24 h. (**e**) Graphical representation of Bax protein levels in MCF-7 and T47D cells, and after treatment with Tam, MSM, or their combination for 24 h

To determine the level of inhibition of human breast adenocarcinoma cell line proliferation mediated by MSM, Tam, and their different combinations, the number of treated cells during the logarithmic phase was compared with that of the non treated control cells. Cell growth was inhibited by ~40 % with 25 μM Tam and ~45 % with 300 mM MSM after 24 h of treatment (Fig. 1a). These concentrations were then used for further experiments. For obtaining the synergic combination dosage, different proportions (1:10000 and 3:40000) of Tam and MSM were used randomly. Compusyn analysis of proliferation inhibition data showed that the combination of Tam and MSM at the 3:40000 ratio had a synergistic effect below Fa = 0.68 (Additional file 1: Table S1). The IC_50_ dosage determined by Compusyn for the combination was 198.619 mM of MSM and 14.9 μM of Tam. This combination showed a synergistic effect with a CI value of 0.51. Therefore, we employed 200 mM of MSM and 15 μM of Tam as the combination concentration for further experiments (Fig. 1b).

### The combination of tamoxifen and MSM induced apoptosis in MCF-7 cells

The proliferation inhibition assay demonstrated that the combination could induce growth arrest. Our next aim was to detect the ability of the combination to induce apoptosis. For detecting and quantifying the cells undergoing apoptosis, we performed annexin V-FITC flow cytometry (Fig. 1c). The cells undergoing necrotic death were counter stained with propidium iodide. 10 μM camptothecin served as a positive control. The obtained results showed that the combination had a stronger ability to induce apoptosis (46 %) than the individual values of Tam (30 %) and MSM (38 %) even though the concentrations of the individual drugs in the combination were lower. An increased Bax expression level provided strong evidence of the induction of apoptosis by the drug combination (Fig. 1d). Our drug combination gave a significant up-regulation of Bax level in total proteins (Fig. 1e). The pro-apoptotic ability of our drug combinations was confirmed by a DNA ladder assay in ER-positive BCa cells (Additional file 2: Figure S1).

### The combination of MSM and tamoxifen synergistically inhibited the Jak2/STAT5b pathway

**Fig. 2:**
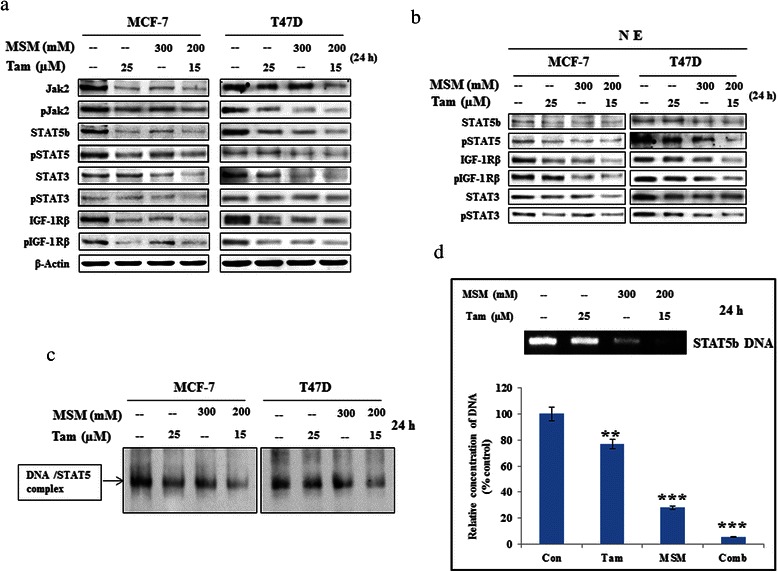
Combination MSM-Tam treatment synergistically inhibited the Jak2/STAT5b signaling pathway. (**a**) Western blotting analysis of cytoplasmic protein levels in MCF-7 and T47D cells, and after treatment with Tam, MSM, or their combination for 24 h. (**b**) Nuclear protein level analysis in MCF-7 and T47D cells, and after treatment with Tam, MSM, or the drug combination for 24 h using western blotting. (**c**) The DNA binding activity of STAT5b was inhibited by the drug combination, as analyzed by a gel shift assay in MCF-7 cells. (**d**) The DNA binding activity of STAT5b was inhibited by the drug combination and confirmed by a ChIP assay in MCF-7 cells. Statistical analyses were conducted using the *ANOVA* test (**P < 0.01 and ***P < 0.001)

The expression levels of different proteins involved in the Jak2/STAT pathway were analyzed by western blotting. As seen in Fig. 2a, combination treatment synergistically inhibited the expression, as well as the phosphorylation, of the Jak2/STAT pathway constituents (Jak2, STAT3, STAT5b, and IGF-1Rβ) in MCF-7 and T47D cells. The combination of Tam and MSM gave an evident result to prove our hypothesis by showing that the levels of expression being downregulated occurred in the setting of the steady expression of the loading control (β actin). This result indicated that Tam and MSM suppressed Jak2, STAT3, STAT5b, and IGF-1Rβ whereas its combination gave more inhibition than individual concentration in both MCF-7 and T47D cells. The densitometrical analysis of Fig. 2a proved the ability of drug combination to down-regulate the tumor proteins (Additional file 3: Figure S2a). In both cell types, however, the signalling molecules were more severely inhibited by the drug combination, even though the concentrations of Tam or MSM in combination were lower.

### The DNA binding activities of STAT5b were inhibited by the drug combination

Phosphorylated STAT3 and STAT5b should be translocated to the nucleus to perform their transcriptional regulation functions. Nuclear translocation was studied using nuclear extracts isolated from MCF-7 and T47D cells pretreated with the combination and the individual agents separately. The western blotting analysis of the nuclear extracts showed a marked decrease in total and phospho STAT5, STAT3, and IGF-1Rβ levels (Fig. 2b) in the combination-treated group as compared to the groups with individual agents (Additional file 3: Figure S2b). The DNA binding activities analyzed using EMSA were confirmed by the ChIP assay (Fig. 2c and d). The obtained results clearly showed that the combination played an important role in the suppression of binding activities.

### The MSM-tamoxifen combination synergistically inhibited downstream targets of the STAT5b pathway

**Fig. 3:**
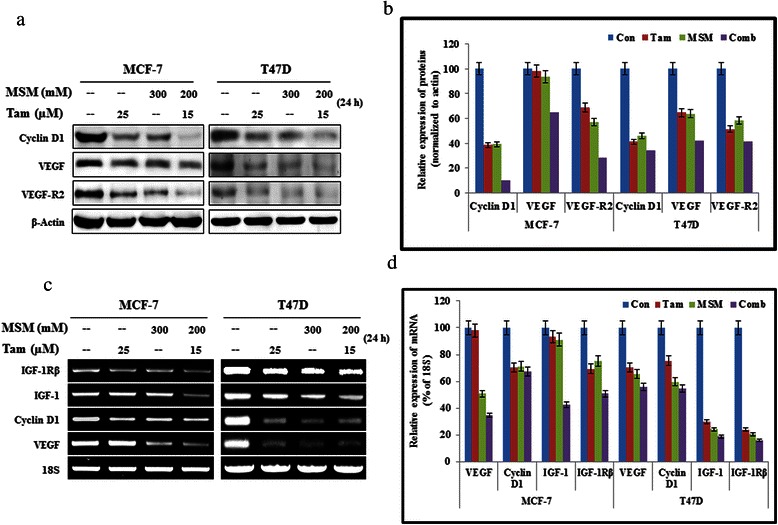
The MSM-Tam combination synergistically inhibited the downstream targets of the STAT5b pathway. (**a**) Western blotting analysis showing total protein levels of the downstream targets of STAT5b following treatment with the drug combination in MCF-7 and T47D cells. (**b**) Graphical analysis of the action of the drug combination and the individual agents on the downstream targets of STAT5b in cytoplasmic proteins. (**c**) RT-PCR analysis of RNA levels of downstream targets of STAT5b after the treatment with Tam, MSM, and the drug combination for 24 h in MCF-7 and T47D cells. (**d**) Inhibition of RNA levels by the drug combination, Tam, and MSM relative to the percentage of 18 s RNA

In the previous section, we found that the MSM-Tam combination synergistically inhibited the STAT5b-DNA binding properties. This inhibition of the DNA binding activities of STAT5b should result in impaired transcription promoter functions. In order to confirm this, the expression of STAT5b downstream targets was analyzed at both the transcriptional (Fig. 3c and d) and translational (Fig. 3a and b) levels. In both cell lines, the expression of cyclin D1, VEGF, IGF-1, and IGF-1Rβ were found to decline in the combination-treated samples (Fig. 3).

### The combination of MSM and tamoxifen synergistically inhibited invasion and migration through STAT5b

**Fig. 4:**
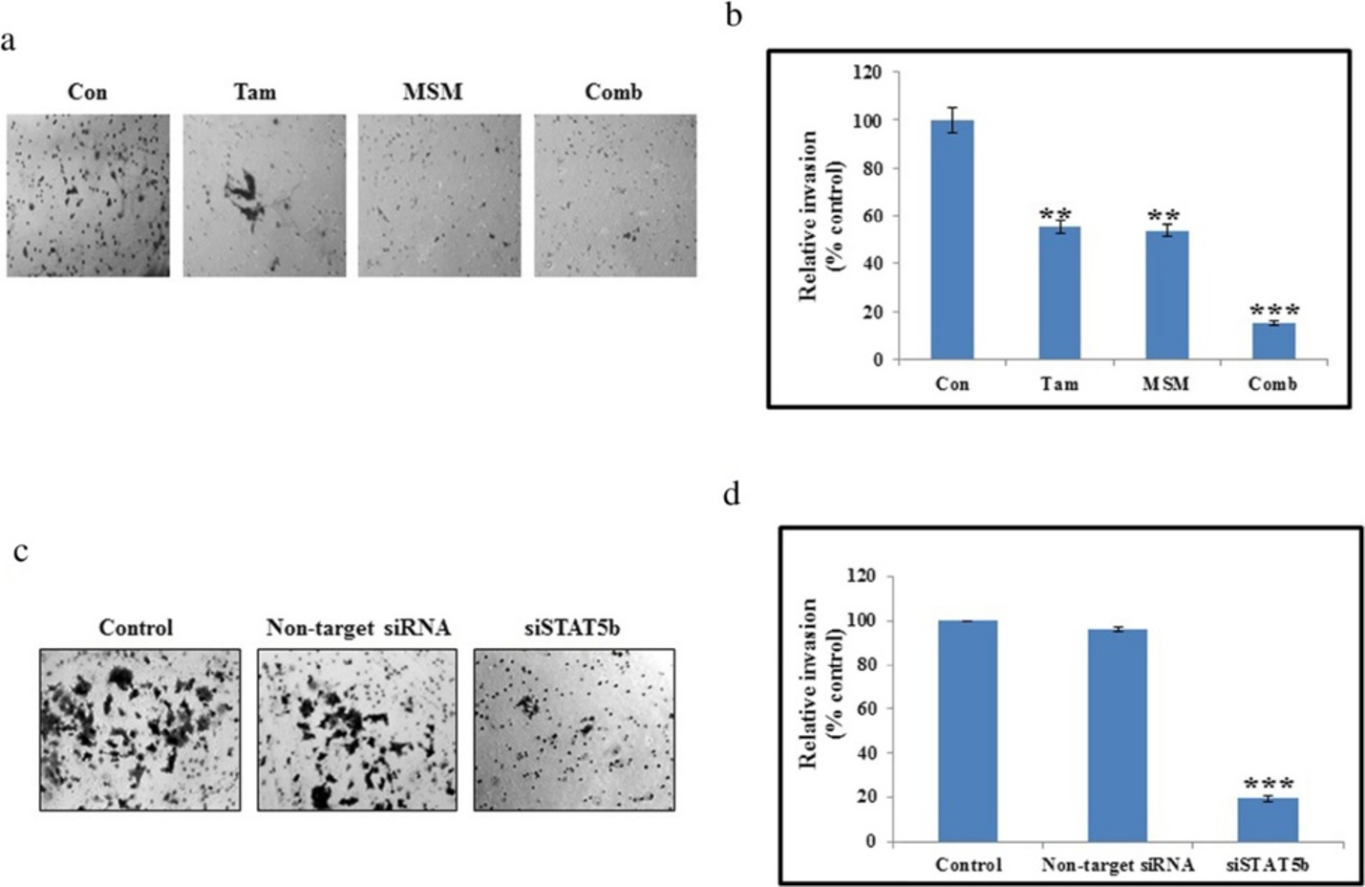
The combination of MSM and Tam synergistically inhibited invasion through STAT5b. (**a**) A Matrigel invasion assay showing the invasion inhibition of Tam, MSM, and the drug combination for 24 h in MCF-7 cells. (**b**) Graphical representation of the invasion assay results. (**c**) On-target inhibition of STAT5b inhibited the invasion in T47D cells. (**d**) Graphical representation of the relative inhibition of invasion after silencing of STAT5b. Statistical analyses were conducted using the *ANOVA* test (**P < 0.01 and ***P < 0.001)

**Fig. 5:**
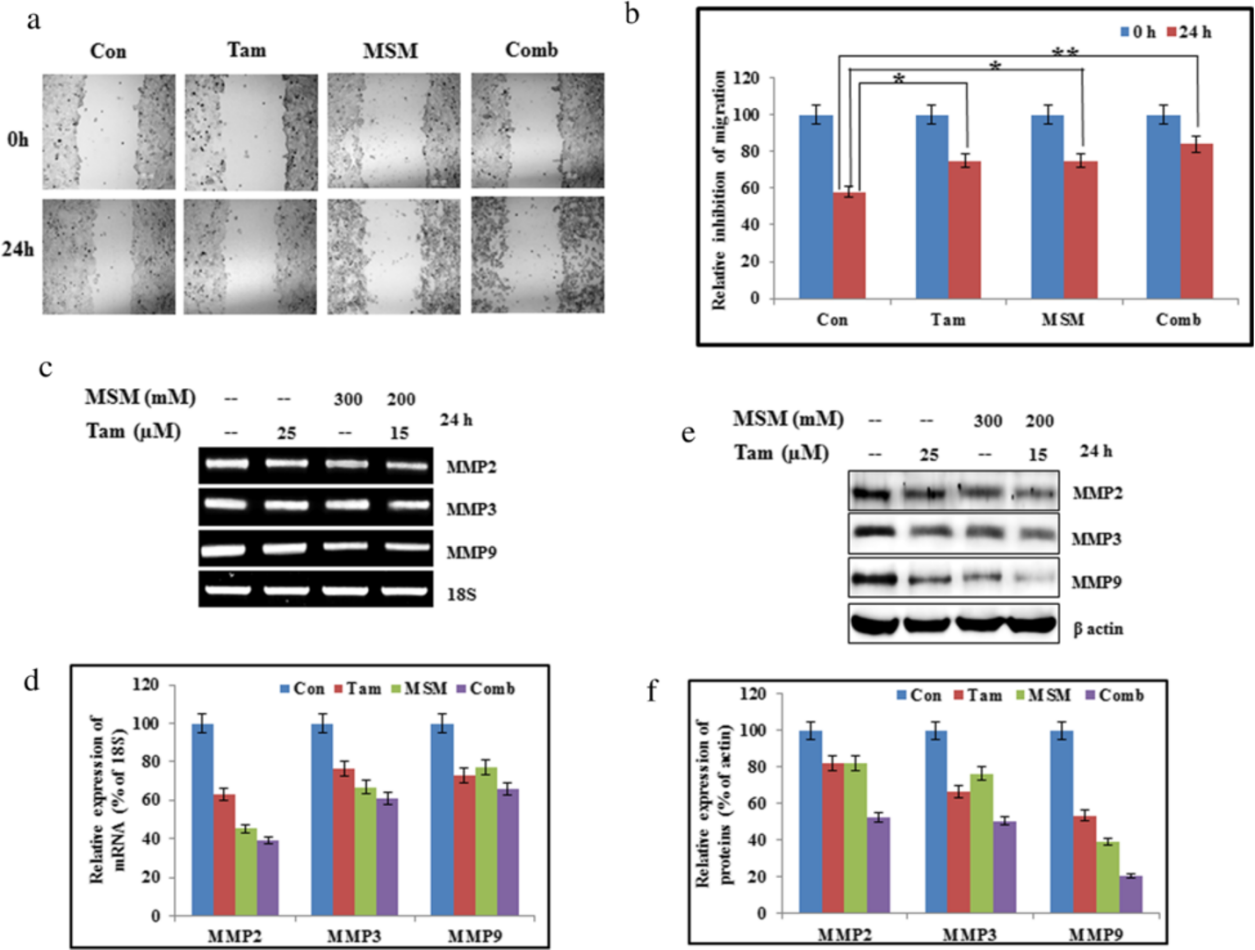
The combination of MSM and Tam synergistically inhibited migration and matrix metalloproteinases. (**a**) A wound healing assay showing the migration inhibition of MCF-7 cells treated with the drug combination and the individual agents for 24 h. (**b**) Relative inhibition of migration in MCF-7 cells as per the wound healing assay. Statistical analyses were conducted using *t*-tests (*P < 0.05 and **P < 0.01). (**c**) RT-PCR analysis of RNA levels of matrix metaloproteins after treatment with Tam, MSM, or the drug combination for 24 h in MCF-7 cells. (**d**) Graphical representation of RNA levels of matrix metaloproteins after treatment with Tam, MSM, or the drug combination for 24 h in MCF-7 cells. (**e**) Western blot analysis showing the levels of matrix metaloproteins in whole cell lysates following treatment with Tam, MSM, or their combination in MCF-7 cells. (**f**) Graphical representation of matrix metaloproteins in whole cell lysates following treatment with Tam, MSM, or their combination in MCF-7 cells

The inhibition of invasion was studied using a Matrigel invasion assay (Fig. 4a). A relatively high level of invasion inhibition was observed in combination-treated cells as compared to those treated with the individual drug concentrations (Fig. 4b, P < 0.01 and P < 0.001). In order to determine the role of STAT5b in invasion, we silenced STAT5b using specific siSTAT5b in T47D cells. Following the silencing of STAT5b, we analyzed the invasion using a Matrigel invasion assay. The obtained result provided strong proof for the role of STAT5b in invasion (Fig. 4c). The use of non-target siRNA showed similar expression levels to those from non-siRNA treated controls. Silenced STAT5b showed a significant invasion inhibition as compared with the non-target group (Fig. 4d). Inhibition of cell migration was determined by an *in vitro* wound healing assay (Fig. 5a). The area of wound closure was quantified using ImageJ software^25^, and the relative inhibition of migration was determined. The results showed a statistically significant inhibition of migration in the combination-treated cells (Fig. 5b, P < 0.05 and P < 0.01). MMPs are the major mediators of invasion *via* digestion of the extracellular membrane, which allows for cancer cells to enter the circulation [37]. Hence, an inhibition in MMP expression should lead to the inhibition of invasion. Our drug combination exerted a synergistic inhibition of MMP2, MMP3, and MMP9 at both the transcriptional and translational levels (Fig. 5c and e). Densitometric analysis of MMPs proved the capability of our drug combination to inhibit invasion (Fig. 5d and f).

### A combination of tamoxifen and MSM inhibited tumor growth

**Fig. 6:**
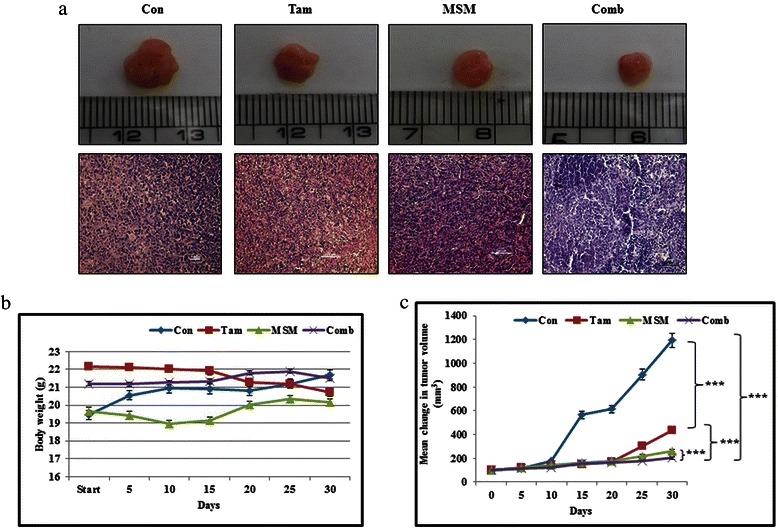
The combination of Tam and MSM inhibited tumor growth. (**a**) Panel 1 represents the xenograft model showing the tumor size obtained following treatment with Tam, MSM, or the drug combination. Panel 2 represents the morphological analysis of tumors by H&E staining. (**b**) Body weight analysis for drug-treated and vehicle-treated mice for a period of 30 days. (**c**) Graphical representation of the tumor size analysis for vehicle-treated controls, and Tam, MSM, or drug combination-treated mice during 30 days of treatment. Statistical analyses were conducted using the *ANOVA* test (***P < 0.001)

The *in vivo* tumor suppressor activity of the drug combination was evaluated in Balb/c nude mice bearing breast tumors induced by MCF-7 cells. After the formation of palpable tumors, mice were treated with the individual drugs and their combination. We observed a statistically significant reduction in tumor volume (Fig. 6c, P < 0.001). The drug combination resulted in a comparatively higher inhibition of tumor growth. The toxicity of the drugs, as evidenced by changes in the weight of the mice, was assessed, and results showed that MSM and the combination treatment had little or no side effects, as there was no reduction in the weight of mice. Conversely, the Tam-treated mice showed a slight decrease in weight (Fig. 6b). The mice were then sacrificed and the xenografts excised for further analysis. Morphological analysis using H&E staining showed a relatively high degree of cell death in the combination treated group (Fig. 6a).

### Inhibition of pulmonary metastasis by the combination of tamoxifen and MSM *in vivo*

**Fig. 7:**
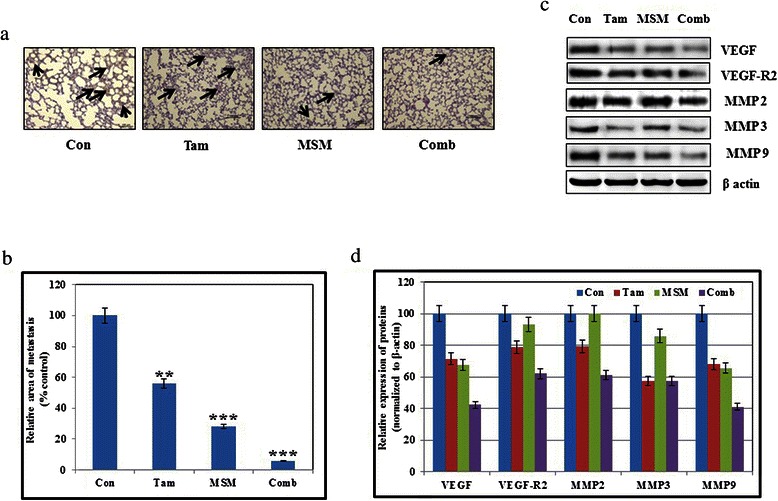
Inhibition of pulmonary metastasis by the combination of Tam and MSM *in vivo.* (**a**) A metastatic animal model showing the pulmonary metastasis analysis for the Tam, MSM, drug combination-treated, or vehicle-treated controls. (**b**) Graphical representation of pulmonary metastasis following treatment with Tam, MSM, or the drug combination with respect to percentage of the control. Statistical analyses were conducted using the *ANOVA* test (**P < 0.01 and ***P < 0.001). (**c**) Tissue protein analysis of various matrix metaloproteins and angiogenic factors after the treatment with Tam, MSM, or the drug combination for 24 h using western blotting. (**d**) Graphical analysis of different tissue proteins following exposure to Tam, MSM, or the drug combination

The *in vitro* analysis revealed that the drug combination had the ability to inhibit the epithelial-mesenchymal transition, as well as the expression of MMPs. Therefore, the ability of the combination to inhibit pulmonary metastasis was analyzed using metastatic animal models. The relative pulmonary metastasis was studied using the lungs excised from the orthotopic animal models (Fig. 7a). The relative metastatic area was detected and plotted with respect to the percentage of metastasis in the controls. Results showed a statistically significant prevention of metastasis by the drug combination (Fig. 7b, P < 0.01 and P < 0.001). The molecular targets for the prevention of pulmonary metastasis were validated *in vivo* using western blotting (Fig. 7c and d). These results showed that the combination inhibited VEGF and VEGF-R2 (responsible for angiogenesis) and MMP2, MMP3, and MMP9 (responsible for invasion).

### Administration of the MSM and tamoxifen combination down-regulated the STAT5b/IGF-1Rβ signaling pathway

**Fig. 8:**
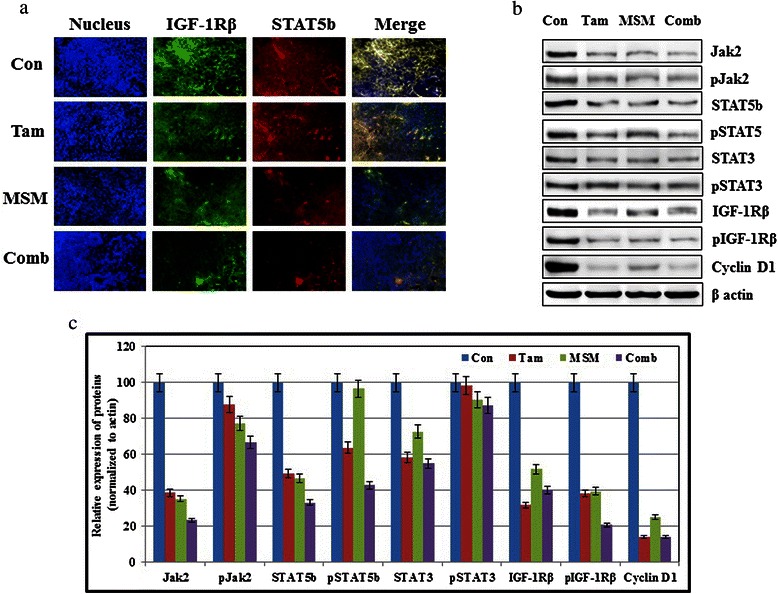
Exposure to the MSM-Tam combination downregulated the STAT5b/IGF-1Rβ signaling pathway. (**a**) Immunofluorescence analysis of STAT5b and IGF-1Rβ showing the inhibition ability of the drug combination. (**b**) Western blotting analysis of various tissue protein levels after treatment with the drug combination or the individual agents for 24 h. (**c**) Relative expression of tissue protein levels following exposure to Tam, MSM, or their combination

In order to elucidate the molecular mechanism by which the drug combination inhibited tumor growth, analyses were performed on xenografts. In theory, the drug combination could have had the capacity to inhibit phosphorylation and activation of STAT5b, and thereby the expression of IGF-1Rβ. The immunofluorescence results showed that treatment with Tam, MSM, and the combination decreased the expression of STAT5b and IGF-1Rβ in the MCF-7 xenograft model without any alteration at the level of the nucleus (Fig. 8a). The western blotting analyses of the tissue protein extracts were concurrent with our previous findings. Additionally, Jak2 and STAT3 were analyzed to assess the involvement of these molecules in tumor growth suppression. Study results clearly demonstrated that the drug combination significantly suppressed the expression and phosphorylation of Jak2, STAT5b, STAT3, and IGF-1Rβ (Fig. 8b and c).

## DISCUSSION

Conventional therapies do not usually have a specific target. Instead, they work *via* the mass killing of cells, which usually results in severe side effects. The advent of combination therapies represents an experimental breakthrough in the use of targeted therapies. A combination of two drugs for the treatment of cancer aims mainly for the reduction of individual drug concentrations while enhancing therapeutic effects. Such combination therapies are multi-targeted and have been shown to be safe and effective in humans.

Tam is well known for its anti-BCa activities by targeting estrogen receptor [38]. The mechanistic role of Tam has been confirmed as the modulation of the STAT5b/IGF-1R pathway, as it acts as an inhibitor of IGF-1, IGF-1Rβ, and STAT5b [39]. However, usage of Tam leads to various critical adverse effects [40]. As such, a great deal of research has been conducted in order to reduce the side effects associated with Tam without reducing its efficacy. Tam used in combination therapy with many other constituents for the treatment of breast cancer [41–43]. It also synergize other drugs in the combination therapy [44, 45] MSM is a natural sulfur containing compound which acts against various breast cancers [10, 27, 28] and is already found as an efficient drug in combination therapy against cancer cells [46]. Combination therapy is one of the methods we can employ to reduce the adverse effects of the drug, either by reducing the concentration of the individual drug, or by synergising the mechanism of the drug. The dosage of MSM we used in this study is a higher concentration. It is not the amount of MSM that contains in food. We used a concentration of 300 mM MSM (individual concentration) just for pharmacological purpose. In order to check the efficacy of combination therapy, we reduced the concentrations of both MSM (200 mM) and Tam (15 μM). Treatment with Tam may cause joint pain to the pateients, whereas MSM is an effective drug for the treatment of joint pain. So the usage of this drug combination may also reduce the joint pain caused by Tam.

The proliferation inhibition ability of the drug combination was determined by an MTT assay (Fig. 1a). The results showed that the different combinations made from Tam and MSM had different degrees of proliferation inhibition ability. The synergistic combination of these two agents was formulated with the help of a Compusyn-based computer simulation (Additional file 1: Table S1). Tis simulation showed that the ratios of 1:10000 and 3:40000 had the ability to inhibit BCa cell proliferation in a synergistic manner. Ideally, anticancer drugs should mediate a maximal rate of cell growth regulation. Hence, we opted to use the ratio of 3:40000 as the synergistic combination for further experiments. The results for apoptosis induction showed that the combination had the ability to induce a maximal rate of apoptosis as compared to the individual agents (Fig. 1c). This was confirmed by DNA strand breaks, which are a hallmark of apoptotic cell death (Additional file 2: Figure S1). Furthermore, the expression of Bax is related to the induction of apoptosis in cells [47]. We observed an increase in the expression of Bax proteins in both the MCF-7 and T47D cells exposed to the combination therapy (Fig. 1d and e), indicating the ability of our drug combination to induce apoptosis.

Jak2 is a receptor kinase known to play a vital role in the Jak2/STAT5b signaling pathway, as activation of Jak2 regulates the activity of downstream molecules in the pathway [48]. Therefore, blockage of Jak2 leads to the blockage of the Jak2/STAT5b pathway. Treatment with the drug combination inhibited Jak2 protein levels *in vitro* and *in vivo*, as well as their phosporylation (Fig. 2a and 8b)*.* STAT5b is the primary substrate of Jak2 [49], which is important in BCa management and takes part in growth hormone signaling. It is a transcription factor that promotes growth and survival of BCa and is considered as a key regulator of tumorigenesis [50]. STAT5b mediates the transcription of numerous genes and is involved in many functions, such as cellular proliferation, differentiation [51, 52], survival [53], cell cycle regulation [54], migration, invasion, and metastasis [55]. We confirmed that the expression of STAT5b and phospho-STAT5 was inhibited by the drug combination at both the cytoplasmic and nuclear levels (Fig. 2a and c).

IGF-1Rβ is highly expressed in BCa [56]. We observed a decrease in IGF-1Rβ at both the cytoplasmic and nuclear levels in cells, as well as *in vivo* when exposed to combination therapy (Fig. 8b). The decrease in IGF-1Rβ can be correlated with the inhibition of DNA binding activity found in the drug combination-treated cells (Fig. 2c and d). The DNA binding of STAT5b is essential for the transcription of downstream targets including IGF-1Rβ [57]. Furthermore, IGF-1Rβ plays important roles in different functions, such as tumor invasion, metastasis [58], and cell death and growth functions [59]. The results of a recent study demonstrated that the inhibition of STAT5b led to a decline in the expression of IGF-1Rβ in BCa cells [10]. This suggests that IGF-1Rβ may be in proportion with STAT5b such that regulation of these molecules is interdependent. Our drug combination showed similar responses for STAT5b and IGF-1Rβ expression. Both transcriptional and translational level inhibition of IGF-1Rβ was observed in cells exposed to the synergistic drug combination. These results were also confirmed *in vivo*. The xenograft model demmonstrated a significant decrease in IGF-1Rβ (Fig. 8a). Both IGF-1Rβ and its phosphorylated form were more reduced by the action of the drug combination than by either of the individual agents alone.

In previous studies, we reporteded that STAT3 was found to be overexpressed in many tumors, especially in BCa [10, 60]. Furthermore, it was shown to have direct associations with many cellular process, such as apoptosis inhibition [61], enhancement of angiogenesis [62], and increasing of metastasis [63]. The obtained results suggested that MSM synergized the activity of Tam in the drug combination by inhibiting the STAT3 molecule and its phosphorylation both *in vitro* and *in vivo*. Nuclear translocation of STAT3 was also found to be decreased by the combination therapy. VEGF is an important downstream target of STAT3 [64], which is involved in the metastasis of BCa *via* increased angiogenesis [65]. The effective inhibition was found in the expression levels of VEGF and its receptor (VEGF-R2) both *in vitro* and *in vivo* after the treatment with drug combination (Fig. 3c and 7c).

The molecular validation of the *in vitro* analysis showed that the combination had the capacity to prevent tumor growth by regulating STAT5b-IGF-1Rβ inhibition and by inhibiting metastasis *via* regulation of the expression of VEGF, VEGF-R2, and the MMPs. This ability was confirmed *in vivo* by BCa xenograft and metastatic animal models. The primary tumor induced in Balb/c athymic nude mice showed a significant decrease in tumor growth in combination-treated animals. Toxicity of the drug and the tumor burden were observed by monitoring dietary habits and body weight gain. The results showed a slight decrease in body weight in Tam-treated animals, while body weight remained unaltered or slightly elevated in all other groups (Fig. 6b). The tumor suppression ability of the drugs was measured by monitoring the volume of tumor. The drug combination provided a statistically significant result after a treatment period of 30 days (Fig. 6c, P < 0.001).

Metastasis is a crucial cause of the mortality in cancer [66], and cell migration and invasion are important steps in metastasis [67]. Hence, inhibition in migration and invasion determines the ability to hinder metastasis. Our drug combination showed a statistically significant inhibition of migration and invasion (Fig. 4b and 5b) as compared to its individual agents. We also proved that STAT5b was the key factor for invasion, and thereby metastasis (Fig. 4c). The STAT5b knock-down showed a statistically significant inhibition in invasion, which demonstrated the role of STAT5b in cell migration and invasion. In order to confirm this, we analyzed the expression levels of MMPs which plays a vital role in cancer metastasis [68–70]. Among MMPs, MMP2 and MMP9 were found to be overexpressed and mediated higher rates of invasion and metastasis in various types of cancers [71–74]. The combination of MSM and Tam inhibited the expression of MMPs *in vitro* and *in vivo* (Fig. 4b and 7c). The expression levels of MMPs and VEGF were down-regulated by drug combination which may be due to the higher rate of apoptosis induction by the combination. But the inhibition of migration, invasion and pulmonary metastasis proved the capability of the drug combination, eventhough it is a weakness of the work in case of animal model. These molecules were downregulated by the action of combination therapy at both the protein and RNA levels (Fig. 5c and d). These results suggest the ability of the drug combination to inhibit migration and invasion, and thereby metastasis. Inhibition of metastasis was confirmed in the metastatic animal model. A significant inhibition of pulmonary metastasis was obtained using the drug combination (Fig. 7a). Only 6 % of all metastases observed was found in the drug combination-treated group (Fig. 7b).

## Conclusions

The results of the current study demonstrated that our drug combination synergistically inhibited the Jak2/STAT5b signaling pathway and also inhibited BCa growth and metastasis, even though the concentrations of the drugs were lower as compared to the individual agents. Therefore, the combination of Tam and MSM may enhance therapeutic efficacy in the treatment of human breast adenocarcinoma. The treatment combination had the added advantage of reducing the dose intensity of the individual drugs, thereby reducing the ocurrence and severity of the adverse effects associated with the use of the individual drugs.

## Additional files

Additional file 1: Table S1. Dose-effect relationships of Tam, MSM and their combinations on growth inhibition of MCF-7 breast adenocarcinoma cells during 24 hours exposure. The parameters *Dm*, *m* and *r* are the slope, antilog of r-intercept, and the linear correlation coefficient of the median-effect plot, which signifies the potency (IC_50_), the shape of the dose-effect curve, and conformity of the data to the mass-action law, respectively. *Dm* and *m* values are used for calculating the CI values. CI < 1, CI =1, and CI > 1 indicate synergism, additivity, and antagonism, respectively. As based on the classic isobologram equation, CI can be calculated by CI = [(D)_1_/(D_x_)_1_] + [(D)_2_/(D_x_)_2_], where D_x_ = Dm[fa/(1 - fa)]^1/m^. The combination ratio was approximately equal to the *Dm* ratio of the component drugs (i.e., close to their equipotency ratio).
Additional file 2: Figure S1. The combination of Tam and MSM induced apoptosis in ER+ breast cancer cells. DNA fragmentation assay showing the ladder formation upon treatment with Tam, MSM, and their combination for 24 h. U937 cells induced apoptosis with camptothecin and were used as positive control.
Additional file 3: Figure S2. (**a**) Densitometrial analysis of cytoplasmic protein levels in MCF-7 and T47D cells, and after treatment with Tam, MSM, or their combination for 24 h. (**b**) Graphical representation of nuclear protein level analysis in MCF-7 and T47D cells, and after treatment with Tam, MSM, or the drug combination for 24 h.

## Abbreviations

Con: Control
Tam: Tamoxifen
MSM: Methylsulfonylmethane
Comb: Combination
NE: Nuclear extract
