# Supplementary material for: The combination of methylsulfonylmethane and tamoxifen inhibits the Jak2/STAT5b pathway and synergistically inhibits tumor growth and metastasis in ER-positive breast cancer xenografts
Source: BMC Cancer. 2015 Jun 19;15:474. doi: 10.1186/s12885-015-1445-0 (PMC4472404; doi:10.1186/s12885-015-1445-0)
Supplement: Additional file 1: Table S1. — Dose-effect relationships of Tam, MSM and their combinations on growth inhibition of MCF-7 breast adenocarcinoma cells during 24 hours exposure. The parameters Dm, m and r are the slope, antilog of r-intercept, and the linear correlation coefficient of the median-effect plot, which signifies the potency (IC50), the shape of the dose-effect curve, and conformity of the data to the mass-action law, respectively. Dm and m values are used for calculating the CI values. CI < 1, CI =1, and CI > 1 indicate synergism, additivity, and antagonism, respectively. As based on the classic isobologram equation, CI can be calculated by CI = [(D)1/(Dx)1] + [(D)2/(Dx)2], where Dx = Dm[fa/(1 - fa)]1/m. The combination ratio was approximately equal to the Dm ratio of the component drugs (i.e., close to their equipotency ratio). [file 12885_2015_1445_MOESM1_ESM.doc]

| **Drugs** | **Dose** | | **Fractional inhibition, *Fa*** | **Dm** | **m** | **r** | **CI** |
| --- | --- | --- | --- | --- | --- | --- | --- |
|  | **Tam (µM)** | **MSM (mM)** |  |  |  |  |  |
| Tam (µM)  [ D_1_] | **15** |  | 0.93 | 26.4145 | 4.8877 | 0.987 |  |
|  | **20** |  | 0.83 |  |  |  |  |
|  | **25** |  | 0.61 |  |  |  |  |
|  | **30** |  | 0.30 |  |  |  |  |
| MSM (mM)  [ **D_2_]** |  | **100** | 0.76 | 393.424 | 0.9218 | 0.963 |  |
|  |  | **200** | 0.70 |  |  |  |  |
|  |  | **300** | 0.56 |  |  |  |  |
|  |  | **400** | 0.47 |  |  |  |  |
| [D_1_] + [D_2_] (1:10000) | **17** | **170** | 0.66 | (0.01844 +184.391) | 8.1627 | 1.000 | 0.46938 |
|  | **20** | **200** | 0.34 |  |  |  |  |
| [D_1_] + [D_2_] (3:40000) | **15** | **200** | 0.48 | (0.01490 +198.619) | 3.6701 | 0.986 | 0.50541 |
|  | **18** | **240** | 0.36 |  |  |  |  |

**Table 1.** Dose-effect relationships of Tam, MSM and their combinations on growth inhibition of MCF-7 breast adenocarcinoma cells during 24 hours exposure. The parameters *Dm*, *m* and *r* are the slope, antilog of r-intercept, and the linear correlation coefficient of the median-effect plot, which signifies the potency (IC_50_), the shape of the dose-effect curve, and conformity of the data to the mass-action law, respectively. *Dm* and *m* values are used for calculating the CI values. CI<1, CI =1, and CI>1 indicate synergism, additivity, and antagonism, respectively. As based on the classic isobologram equation, CI can be calculated by CI = [(D)_1_/(D_x_)_1_] + [(D)_2_/(D_x_)_2_], where D_x_ = Dm[fa/(1 - fa)]^1/m^. The combination ratio was approximately equal to the *Dm* ratio of the component drugs (i.e., close to their equipotency ratio).
