# Supplementary figures and images for: The combination of methylsulfonylmethane and tamoxifen inhibits the Jak2/STAT5b pathway and synergistically inhibits tumor growth and metastasis in ER-positive breast cancer xenografts
Source: BMC Cancer. 2015 Jun 19;15:474. doi: 10.1186/s12885-015-1445-0 (PMC4472404; doi:10.1186/s12885-015-1445-0)

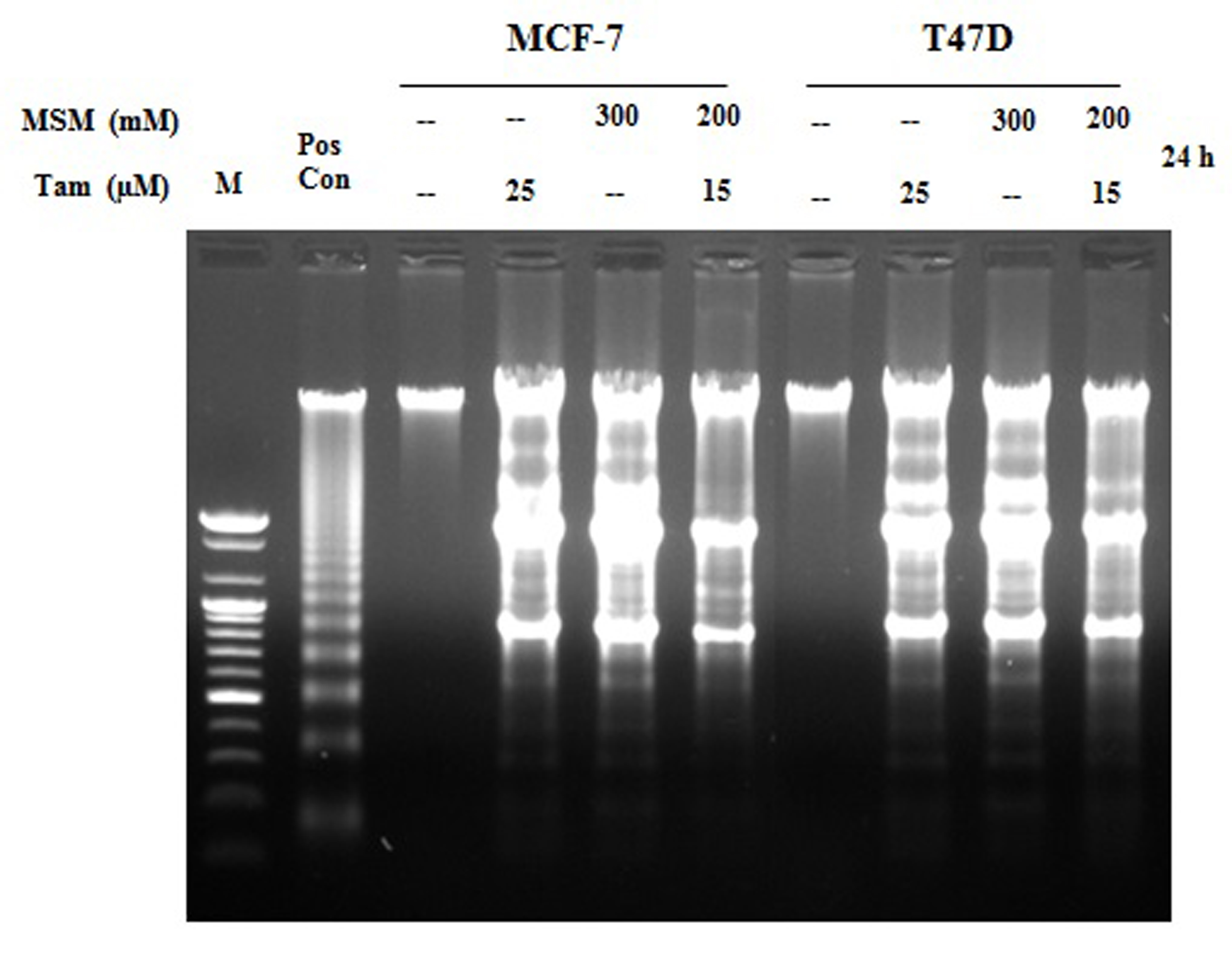

Supplement: Additional file 2: Figure S1. — The combination of Tam and MSM induced apoptosis in ER+ breast cancer cells. DNA fragmentation assay showing the ladder formation upon treatment with Tam, MSM, and their combination for 24 h. U937 cells induced apoptosis with camptothecin and were used as positive control. [file 12885_2015_1445_MOESM2_ESM.jpeg]

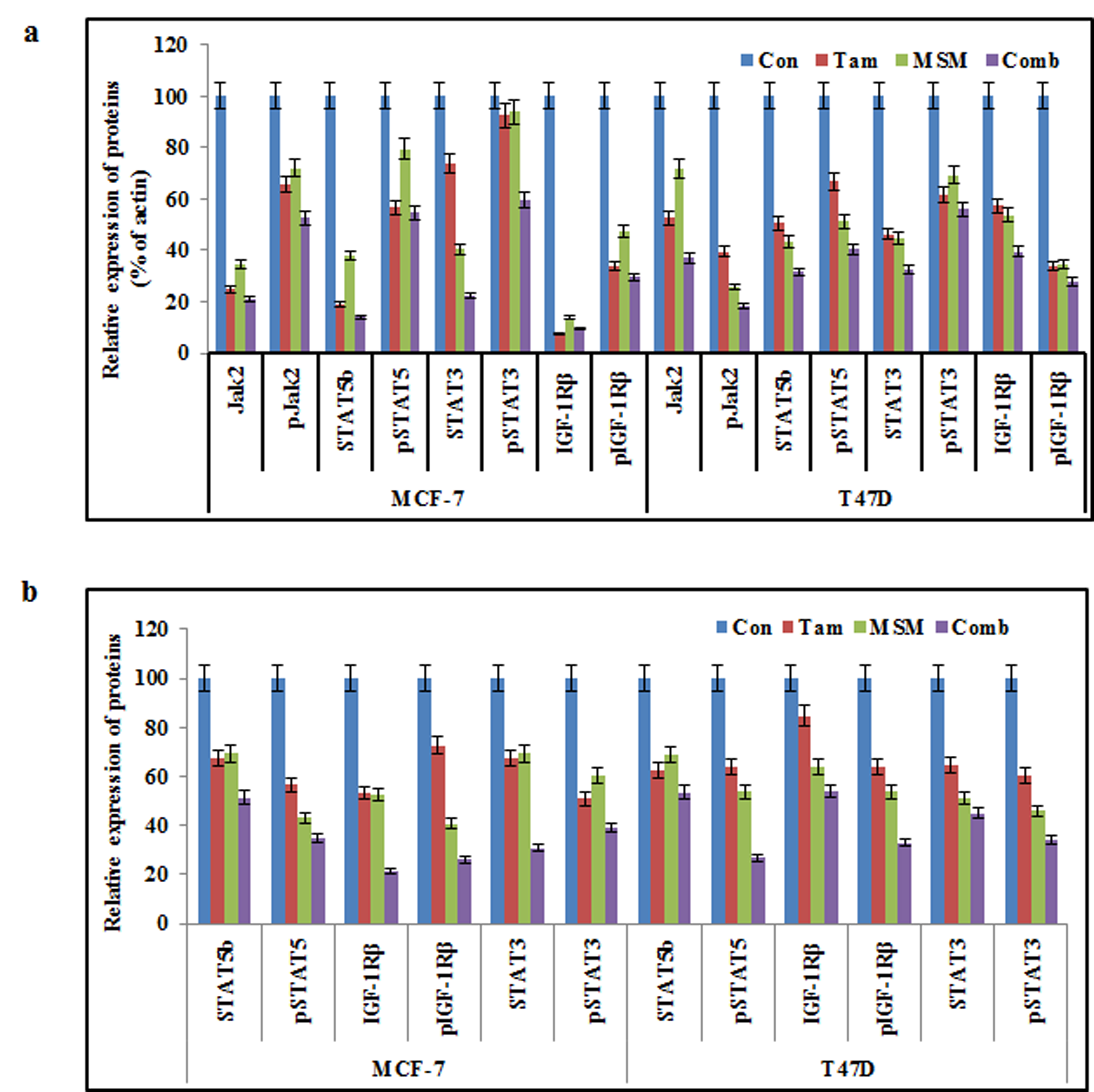

Supplement: Additional file 3: Figure S2. — (a) Densitometrial analysis of cytoplasmic protein levels in MCF-7 and T47D cells, and after treatment with Tam, MSM, or their combination for 24 h. (b) Graphical representation of nuclear protein level analysis in MCF-7 and T47D cells, and after treatment with Tam, MSM, or the drug combination for 24 h. [file 12885_2015_1445_MOESM3_ESM.jpeg]
